# Impact of diurnal temperature and relative humidity hysteresis on atmospheric dryness in changing climates

**DOI:** 10.1126/sciadv.adu5713

**Published:** 2025-06-27

**Authors:** Ching-Hung Shih, Yi-Shin Jang, Tzu-Ying Yang, Cho-Ying Huang, Jehn-Yih Juang, Min-Hui Lo

**Affiliations:** ^1^ Department of Atmospheric Sciences, National Taiwan University, Taipei, Taiwan.; ^2^ Department of Geography, National Taiwan University, Taipei, Taiwan.

## Abstract

Vapor pressure deficit (VPD), a key indicator of atmospheric dryness, is strongly influenced by diurnal cycles of temperature (*T*) and relative humidity (RH). While these cycles are typically inversely locked in-phase, recent research has identified diurnal hysteresis, characterized by a time lag between *T* and RH; yet, its impact on VPD under changing climates remains poorly understood. In this study, we examine how diurnal *T*/RH hysteresis modulates VPD across different climates using observational data alongside high-resolution reanalysis and simulations. Here, we find that regions exhibiting strong diurnal *T*/RH hysteresis, especially in some waterside and montane regions, experience earlier daily VPD peaks. We also demonstrate that global warming weakens diurnal *T*/RH hysteresis, leading to amplified VPD increasing trends and greater ecosystem stress. These results highlight the need for improved representation of diurnal *T*/RH interactions in climate models to better predict atmospheric dryness and its impacts on land-atmosphere feedbacks, ecosystems, and regional water cycles.

## Teaser

An out-of-phase diurnal cycle of temperature and relative humidity modulates atmospheric dryness across different climates.

## INTRODUCTION

Land-atmosphere processes are often characterized by pronounced diurnal cycles of meteorological (e.g., net radiation) and ecological [e.g., evapotranspiration (ET)] variables that exhibit in-phase relationships, which can be either positively or inversely correlated with one another (*1*–*3*), yet not all locations follow these characteristics. Recent studies have focused on “out-of-phase” characteristics, also known as diurnal hysteresis. For example, past studies have documented diurnal hysteresis between evapotranspiration and meteorological variables, such as vapor pressure deficit (VPD) and temperature (*T*), with its magnitude influenced by factors such as soil water availability (abiotic), leaf and root water potentials (biotic), and the time lag between radiation and VPD (*4*–*7*). Because the magnitude of diurnal hysteresis results from nonlinear interactions between variables during the morning and afternoon, analyzing these relationships offers valuable insights into the factors controlling land-atmosphere processes on the subdaily scale (*7*, *8*).

Among the variables examined for diurnal hysteresis, VPD, the difference between the saturated and actual vapor pressure, is often used to guide ecological discussion and assess evapotranspiration demands. Higher VPD increases atmospheric water demand, thereby enhancing potential evapotranspiration (*9*). However, under hot and dry conditions, higher VPD can reduce transpiration as plants close their stomata to conserve water, thus decreasing evapotranspiration (*10*). Such opposing effects of VPD result in diverse evapotranspiration responses across different climates and plant types (*11*, *12*). Because VPD is directly affected by diurnal cycles of *T* and relative humidity (RH), variations in these cycles directly influence VPD and subsequent evapotranspiration. Therefore, analyzing the subdaily dynamics between *T* and RH is crucial for understanding VPD, evapotranspiration, and other land-surface processes (*1*, *3*).

Diurnal hysteresis between *T* and RH (abbreviated hereafter as diurnal *T*/RH hysteresis) has been overlooked. This oversight may arise from the assumption that diurnal variations in *T* and RH are tightly coupled in an inverse, in-phase relationship (*13*), primarily driven by the effect of *T* fluctuations on RH. However, observational data from the montane cloud forests of northeastern Taiwan revealed clear diurnal *T*/RH hysteresis, with RH increasing as *T* rises in the morning. This is likely due to water vapor contributions from mountain-valley winds and local evapotranspiration (*14*). While this finding is based on a specific region, it underscores the potential role of local processes in shaping diurnal *T*/RH hysteresis. Such a phenomenon could be more widespread than previously recognized, affecting local hydrological cycles and evapotranspiration partitioning (*14*, *15*). These considerations motivate the need for a global assessment of diurnal *T*/RH hysteresis.

The diurnal ranges and daily courses of *T* and RH are expected to change under global warming. One well-documented trend is the global asymmetry in diurnal warming, where nighttime *T* increases more rapidly than daytime *T*, reducing the diurnal *T* range (*16*, *17*). However, this trend is not uniform, with some regions experiencing an increase in diurnal *T* range (*18*). These *T* changes subsequently influence the diurnal specific humidity range, as a warmer atmosphere has a greater capacity to hold moisture (*19*). In addition, shifts in atmospheric circulation patterns because of global warming may further modify the diurnal cycle of specific humidity (*20*). Despite these observations, the impact of global warming on diurnal *T*/RH hysteresis, and by extension, its influence on the diurnal cycle of VPD, remains unclear and warrants further investigation.

To address these knowledge gaps, our research begins by examining two distinct sites, including Chi-Lan (TW-CLM) [1650 m above sea level (a.s.l.), cloud forests] and Lien-Hua-Chih (TW-LHC) (780 m a.s.l., non–cloud forests) sites in Taiwan (fig. S1), which exhibit different diurnal relationships between *T* and RH. We use a multilinear regression approach to determine the phase lag time ( $t_{\phi }$ ) between the diurnal cycles of *T* and RH (see Materials and Methods) and analyze the causes of the differences between the two sites. Subsequently, we extend our investigation globally to document the regions exhibiting diurnal *T*/RH hysteresis by analyzing observational datasets, including Met Office Hadley Centre Integrated Surface Database (HadISD) (*21*–*24*) and FLUXNET2015 (*25*), as well as the ERA5-Land reanalysis dataset (*26*). In the following sections, we focus on ERA5-Land results from boreal summer [June, July, and August (JJA)], with findings from boreal winter [December, January, and February (DJF)] provided in the Supplementary Materials. To assess the impacts of global warming on diurnal *T*/RH hysteresis, we also apply a pseudo-global warming (PGW) approach (see Materials and Methods) to investigate how diurnal *T*/RH hysteresis might change under different levels of global warming.

## RESULTS

### Current distribution of diurnal *T*/RH hysteresis

**Fig. 1.**
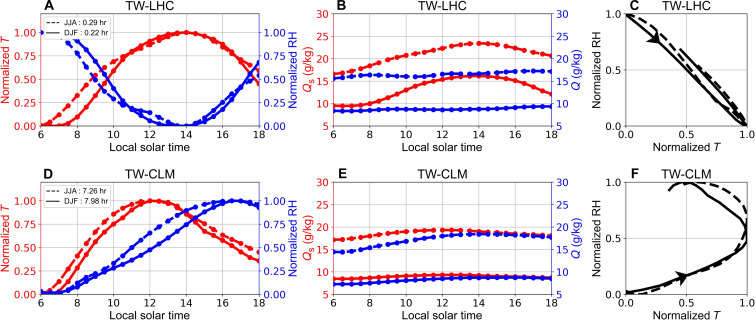
Comparison of the diurnal cycle at TW-LHC from 2009 to 2011 and TW-CLM from 2008 to 2011 in Taiwan. (**A** and **D**) Diurnal cycle of normalized *T* (red lines) and RH (blue lines) at the TW-LHC and TW-CLM sites, with JJA (dashed lines) and DJF (solid lines) indicating seasonal variations. Note that *T* and RH are normalized by subtracting the minimum value of the feature from each value and then dividing by the range of the feature. The legend denotes the phase lag time ( $t_{\phi }$ ) between *T* and RH in different seasons. hr, hours. (**B** and **E**) Diurnal cycle of saturated specific humidity (*Q*_s_; red lines) and specific humidity (*Q*; blue lines) at the TW-LHC and TW-CLM sites, with JJA (dashed lines) and DJF (solid lines) indicating seasonal variations. (**C** and **F**) Diurnal phase diagram between normalized *T* and RH in JJA (dashed lines) and DJF (solid lines) in TW-LHC and TW-CLM, respectively. All hysteretic cycles are counterclockwise. Note that the mean diurnal curves are derived by spline fitting to the half-hourly data.

Using a multilinear regression approach for examining the diurnal cycle of *T* and RH, our analysis reveals that in JJA and DJF, *T* at TW-LHC lags behind RH by 0.29 and 0.22 hours, respectively, while at TW-CLM, this lag extends to 7.26 and 7.98 hours (Fig. 1, A and D). The diurnal phase diagrams further illustrate the differences between TW-LHC and TW-CLM. While TW-LHC exhibits a nearly linear relationship between diurnal *T* and RH, TW-CLM shows a broader hysteretic loop, with diurnal RH increasing as *T* rises in the morning (Fig. 1, C and F). The increase in RH can be attributed to the rapidly increasing specific humidity and slowly increasing saturated specific humidity in daytime in TW-CLM compared with TW-LHC (Fig. 1, B and E). The diurnal variations in RH at TW-CLM are influenced not only by *T* variations (Eq. 1) but also by diurnal changes in water vapor. The changes in water vapor may result from a rapid increase in morning evapotranspiration and moisture transport from lower elevation regions driven by mountain-valley winds throughout the day, in contrast to conditions observed at TW-LHC (*14*).

**Fig. 2.**
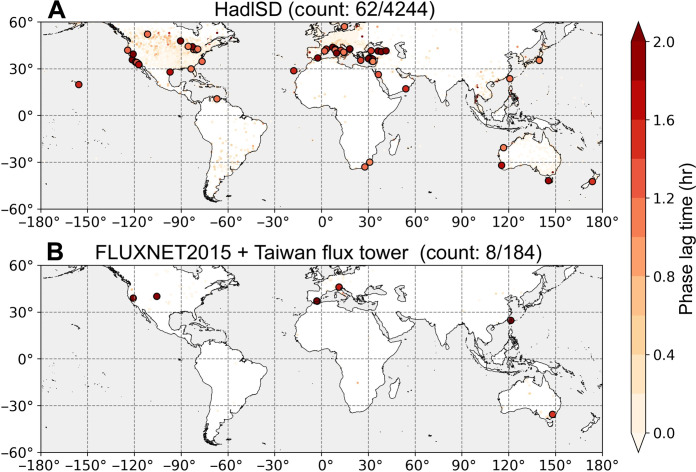
Presence of diurnal *T*/RH hysteresis across global weather stations and flux tower sites. (**A** and **B**) $t_{\phi }$ (hours) of HadISD weather stations and flux tower sites, respectively. The color of the dots illustrates $t_{\phi }$ (hours) between *T* and RH. Stations exhibiting diurnal *T*/RH hysteresis are defined as those where $t_{\phi }$ exceeds 1 hour, shown as larger dots in (A) and (B). The counts noted in the title of (A) and (B) represent the number of stations exhibiting diurnal *T*/RH hysteresis out of the total number of stations.

We then identify the presence of diurnal *T*/RH hysteresis (with $t_{\phi }$ exceeding 1 hour) globally using data from 4244 HadISD weather stations and 184 flux tower sites, including FLUXNET2015 and Taiwan flux towers. Globally, less than 5% of the studied sites exhibit $t_{\phi }$ greater than 1 hour; thus, the majority is more similar to the TW-LHC site. Specifically, 62 stations in the HadISD dataset and 8 stations in the flux tower dataset exhibit diurnal *T*/RH hysteresis. These sites are mostly located in montane regions, such as Taiwan and the Alps, or near water bodies, including the Great Lakes and coastal regions (larger dots in Fig. 2). In addition, weak signals of diurnal *T*/RH hysteresis, with $t_{\phi }$ ranging from half an hour to 1 hour, are found in North America, the European Plain, and Peninsular Southeast Asia, represented by smaller colored dots in Fig. 2.

**Fig. 3.**
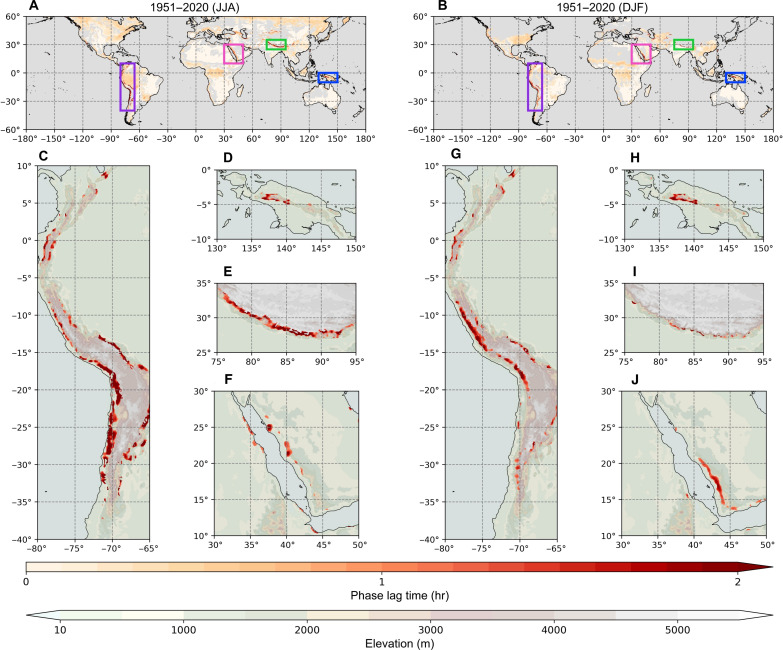
Presence of diurnal *T*/RH hysteresis in ERA5-Land from 1951 to 2020. Presence in (**A** and **C** to **F**) JJA and (**B** and **G** to **J**) DJF. The boxes illustrate the main four regions exhibiting diurnal *T*/RH hysteresis, including the Andes (purple boxes) [(C) and (G)], New Guinea (blue boxes) [(D) and (H)], the Himalayas (green boxes) [(E) and (I)], and Southwestern Arabia (pink boxes) [(F) and (J)]. The red monochromatic colors in all panels represent $t_{\phi }$ (hours). The gray shadings represent the grids that were masked because of the significant test and *T* lower than 0°C. The green-brown-white color gradient in (C) to (J) represents the elevation. Note that only grids exhibiting diurnal *T*/RH hysteresis, defined as those where $t_{\phi }$ exceeds 1 hour, are shown in (C) to (J).

To overcome the spatial limitations of observational datasets, particularly in regions with insufficient hourly observations, such as South America and the Maritime Continent, we use ERA5-Land, a high-resolution land reanalysis dataset, to conduct a global analysis on diurnal *T*/RH hysteresis. ERA5-Land confirms the presence of diurnal *T*/RH hysteresis in some montane and waterside regions observed in the observational dataset, such as Taiwan, the Alps, and the Great Lakes, but also identifies additional regions, including the montane areas of New Guinea, southwestern Arabia, the Andes, and the Himalayas, as having strong diurnal *T*/RH hysteretic relationships across both seasons. These findings suggest a higher likelihood of diurnal *T*/RH hysteresis occurring within mountainous areas with elevations ranging from ~800 to 3600 m a.s.l. (Fig. 3).

### Current diurnal characteristics in regions with diurnal *T*/RH hysteresis

**Fig. 4.**
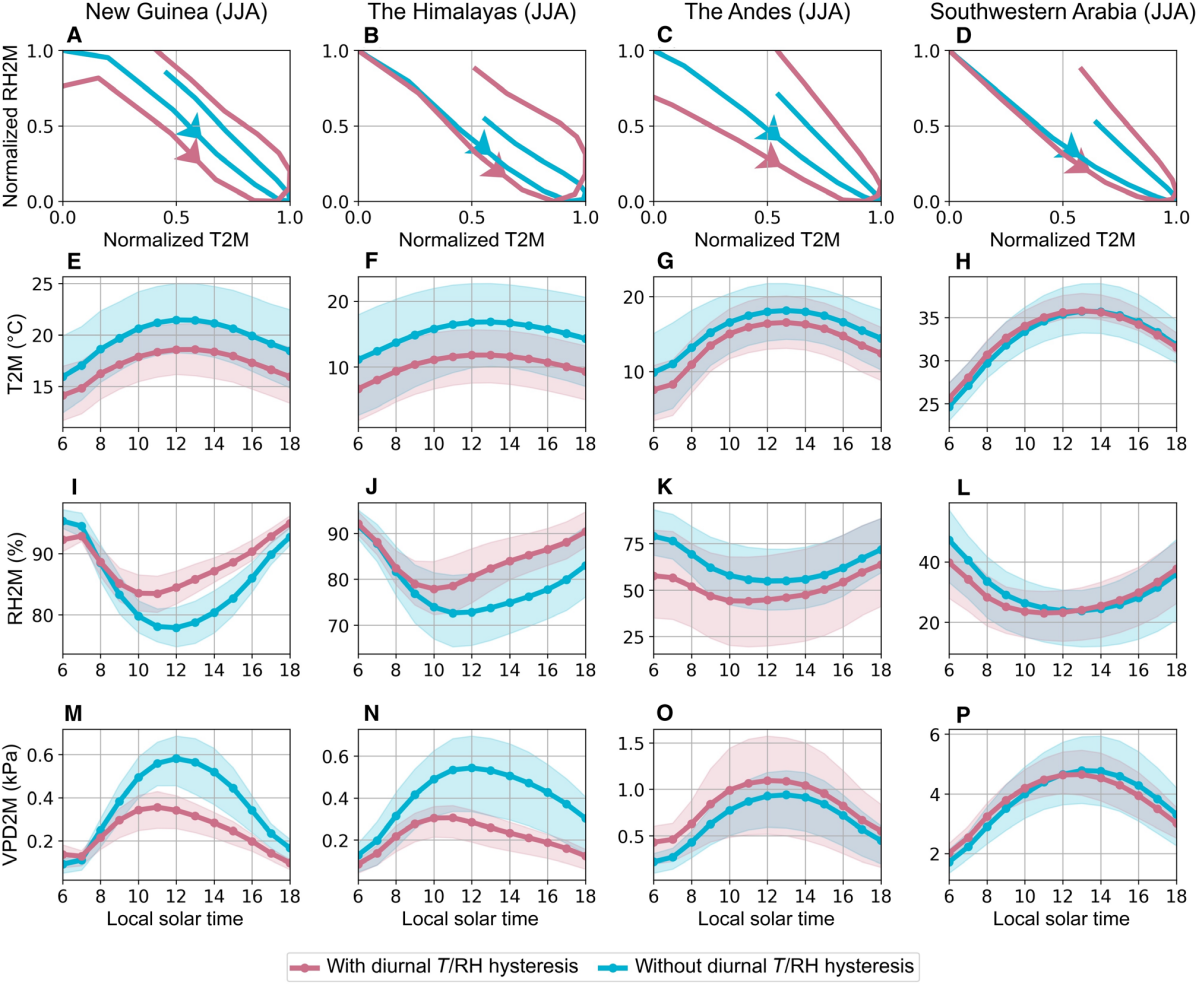
Diurnal cycles from 1951 to 2020 in areas exhibiting diurnal *T*/RH hysteresis (red lines) and adjacent areas without diurnal *T*/RH hysteresis (blue lines) across the four diurnal *T*/RH hysteresis hotspots. (**A** to **D**) Diurnal phase diagram between normalized *T* and normalized RH. All hysteretic cycles are counterclockwise. Diurnal cycle of (**E** to **H**) 2-m *T* (T2M; °C), (**I** to **L**) 2-m RH (RH2M; %), and (**M** to **P**) 2-m VPD (VPD2M; kPa). The shadings represent the range of variation of each meteorological variable between the first and third quartiles of data. It is noteworthy that the surrounding areas without diurnal *T*/RH hysteresis (blue lines) were selected through buffer analysis. This analysis ensured that the number of grid cells without diurnal *T*/RH hysteresis approximates the number of those with diurnal *T*/RH hysteresis, facilitating a balanced comparison between regions with and without this phenomenon. Note that the mean diurnal curves are derived by spline fitting to the hourly data.

We further investigate the distinct diurnal characteristics in the ERA5-Land dataset by comparing the diurnal cycles between areas having diurnal *T*/RH hysteresis, where $t_{\phi }$ is more than 1 hour, and their adjacent areas lacking such diurnal *T*/RH hysteresis. In the diurnal phase diagrams, the width of the hysteretic loops in areas exhibiting diurnal *T*/RH hysteresis is larger than that in adjacent areas for both JJA and DJF seasons (Fig. 4, A to D, and fig. S2, A to D).

Figure 4 (E to L) and fig. S2 (E to L) further illustrate that the diurnal *T*/RH hysteresis primarily results from the earlier occurrence of the minimum RH in areas exhibiting diurnal *T*/RH hysteresis compared to adjacent areas. Specifically, in all areas of these four regions, *T* reaches its maximum between 12:00 p.m. and 2:00 p.m. (local time hereafter) regardless of the presence of diurnal *T*/RH hysteresis. However, in areas exhibiting diurnal *T*/RH hysteresis, RH reaches its minimum between 10:00 a.m. and 12:00 p.m., which differs from the diurnal cycle of RH in the adjacent areas.

In regions exhibiting an in-phase relationship between *T* and RH, VPD reaches daily maximum when *T* peaks, coinciding with the minimum RH. However, in regions exhibiting diurnal *T*/RH hysteresis, this hysteretic relationship can mitigate VPD because of the differing timing of maximum *T* and minimum RH (Fig. 4, M to P, and fig. S2, M to P). The diurnal cycle of VPD shows a misalignment with the diurnal cycle of *T* in the four regions. Specifically, in New Guinea and the Himalayas, diurnal *T*/RH hysteresis shifts the daily maximum VPD between 10:00 a.m. to 12:00 p.m., aligning with the diurnal cycle of RH. In the Andes and southwestern Arabia, diurnal *T*/RH hysteresis slightly shifts the daily maximum VPD to occur earlier than the maximum *T*. The VPD mitigation effect induced by diurnal *T*/RH hysteresis not only modulates overall VPD levels and but also alters the diurnal cycle of VPD.

**Fig. 5.**
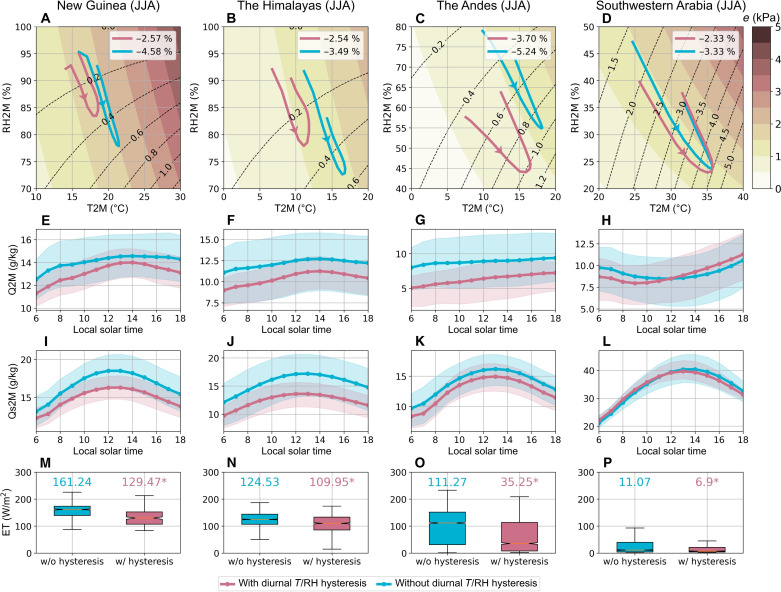
Comparison between areas exhibiting diurnal *T*/RH hysteresis (red) and adjacent areas without diurnal *T*/RH hysteresis (blue) from 1951 to 2020 across the four diurnal *T*/RH hysteresis hotspots. (**A** to **D**) Regional mean of the diurnal phase diagram between 2-m *T* (T2M) and 2-m RH (RH2M). The shading within each panel indicates vapor pressure (kPa), while black dashed lines denote VPD (kPa). The legends quantify the bias in mean daytime (from 6:00 a.m. to 6:00 p.m.) VPD estimation (%). All hysteretic cycles are counterclockwise. Diurnal cycle of (**E** to **H**) 2-m specific humidity (Q2M; g/kg) and (**I** to **L**) 2-m saturated specific humidity (Qs2M; g/kg). The shadings represent the range of variation of each meteorological variable between the first and third quartiles of data. (**M** to **P**) Comparison of evapotranspiration (W/m^2^) from 6:00 a.m. to noon. The numbers above the boxes denote the median values. The presence of evapotranspiration (ET) is significantly lower in these hysteresis regions. These findings are supported by Student’s *t* tests, with asterisks (*) denoting *P* values smaller than 0.05 (see Materials and Methods for the definition of bias in mean daytime VPD estimation). Note that the mean diurnal curves in (E) to (L) are derived by spline fitting to the hourly data.

In addition, the VPD mitigation effect can influence the accuracy of daily VPD estimates. Typically, daily VPD is underestimated when calculated using mean daytime *T* and RH from 6:00 a.m. to 6:00 p.m. because of the nonlinear relationship between saturated vapor pressure and *T* based on the Clausius-Clapeyron equation (see Materials and Methods). However, regions with diurnal *T*/RH hysteresis show significantly smaller estimation errors (unpaired one-tailed Student’s *t* test; *P* < 0.01), specifically a 2 to 4% underestimation of daily VPD, compared to adjacent areas without hysteresis, which exhibit a 3 to 6% underestimation (Fig. 5, A to D, and fig. S4, A to D). This reduction in error stems from the effect of diurnal *T*/RH hysteresis in modulating daily maximum VPD.

To investigate the mechanism behind the formation of diurnal *T*/RH hysteresis, we further examine the diurnal cycle of specific humidity (*Q*; representing the water vapor effect on RH) and saturated specific humidity (*Q*_s_; representing the *T* effect on RH). Because *T* reaches its maximum between 12:00 p.m. and 2:00 p.m. in both regions with and without diurnal *T*/RH hysteresis, the rate of increase in daytime *Q*_s_ is similar across these areas (Fig. 5, I to L, and fig. S4, I to L). In contrast, regions with diurnal *T*/RH hysteresis exhibit a larger increase in daytime *Q* (Fig. 5, E to H, and fig. S4, E to H), which is the primary factor driving the diurnal *T*/RH hysteresis pattern. This larger morning *Q* range is also reflected in broader hysteretic loops in the diurnal phase diagrams, where the major axis of the diurnal *T*-RH cycle aligns closely with the isolines of vapor pressure (Fig. 5, A to D, and fig. S4, A to D). However, smaller evapotranspiration during morning is observed in some regions exhibiting diurnal *T*/RH hysteresis (Fig. 5, I to L, and fig. S4, I to L), indicating that a nonlocal moisture source can play a role in increasing morning *Q*.

### Diurnal *T*/RH hysteresis in a warming world

**Fig. 6.**
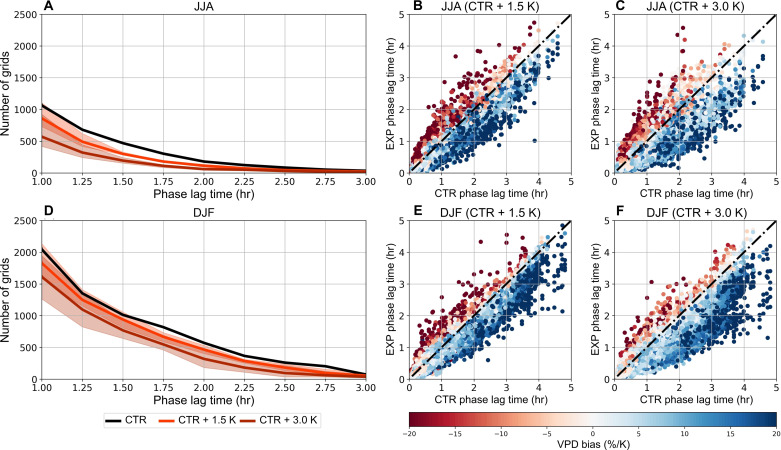
Change of diurnal *T*/RH hysteresis in different warming scenarios of PGW simulations in the Andes regions. (**A** and **D**) Presence of diurnal *T*/RH in JJA and DJF, respectively. The black lines denote the distribution under CTR. The light red and dark red lines represent the mean distribution of six RCM-driven PGW simulations under global mean surface *T* increases by 1.5 and 3.0 K, respectively. Relative change of VPD bias to *T* (%/K) in (**B** and **C**) JJA and (**E** and **F**) DJF in each grid of six RCM-driven PGW simulations [experimental run (EXP)]. The blue dots denote an increase in VPD underestimation bias, while the red dots denote a decrease in VPD underestimation bias.

We further investigate the impact of global warming on diurnal *T*/RH hysteresis, focusing on the Andes region, which has experienced a significant decrease in diurnal *T*/RH hysteresis in both seasons in recent years (table S1). Community Land Model version 5 (*27*) (CLM5) is used to assess the impact of global warming on diurnal *T*/RH hysteresis under Representative Concentration Pathway 8.5 scenarios, with global mean surface *T* increases of 1.5 and 3.0 K, relative to the baseline period from 1986 to 2005 (see Materials and Methods). Figure 6 (A and D) reveals a noticeable contraction in areas exhibiting diurnal *T*/RH hysteresis in the Andes, particularly for $t_{\phi }$ greater than 2 hours, with all regional climate model (RCM)–driven simulations showing a reduction. This suggests that most grid areas previously exhibiting diurnal *T*/RH hysteresis in the control simulations transition to an in-phase relationship between *T* and RH, highlighting the pronounced impact of global warming on diurnal *T*/RH hysteresis patterns in these regions.

Figure 6 (B, C, E, and F) shows that the relative change in VPD bias to *T* is directly linked to changes in the diurnal *T*/RH hysteretic relationship in the Andes. Specifically, areas with enhanced diurnal *T*/RH hysteresis under global warming show a reduced VPD underestimation bias relative to *T* change because of increased VPD mitigation effects. Conversely, areas with reduced $t_{\phi }$ exhibit an enlarged VPD underestimation bias relative to *T* change because of the diminished *T*/RH hysteresis. In addition, greater deviations from the one-to-one line, which are associated with larger changes in $t_{\phi }$ , exhibit more pronounced VPD biases relative to *T* response. This suggests that the extent of changes in diurnal *T*/RH hysteresis directly influences the VPD mitigation effect and consequently affects the accuracy of VPD estimation. Under global warming, the underestimation error in daily VPD may increase in most of regions currently exhibiting diurnal *T*/RH hysteresis.

## DISCUSSION

Diurnal *T*/RH hysteresis, characterized by an “out-of-phase” relationship between daytime *T* and RH, is observed in certain montane and coastal regions (Figs. 2 and 3). This phenomenon is found in less than 5% of all observation sites (combining HadISD and flux tower data), with only 1.46% of stations in the HadISD dataset exhibiting it. This limited occurrence may be largely due to the fact that most weather stations are not located in the montane and coastal regions, where ERA5-Land data indicate its presence. Despite its limited spatial extent, diurnal *T*/RH hysteresis is particularly pronounced in ecologically critical areas, such as cloud forests and fog-dependent ecosystems, as identified in the ERA5-Land dataset. Cloud forests, forest ecosystems frequently immersed in clouds and fog, are present in regions such as Taiwan, the Andes, and New Guinea (*28*–*30*), all of which exhibit diurnal *T*/RH hysteresis in our analysis. These ecosystems host exceptionally high levels of endemism and numerous threatened species, making them globally recognized biodiversity hotspots (*28*, *31*). The occurrence of diurnal *T*/RH hysteresis in these regions suggests a potential link between this unique diurnal characteristic and the functioning of vulnerable ecosystems. Similarly, fog-dependent ecosystems, including those in southwestern Arabia and certain coastal regions (*32*, *33*), rely heavily on fog as a primary water source for sustaining vegetation and wildlife, particularly in water-limited environments. Understanding the formation of diurnal *T*/RH hysteresis and its potential shifts under future climate change is therefore essential for predicting hydrological changes and assessing ecosystem resilience in these biodiversity hotspots.

**Fig. 7.**
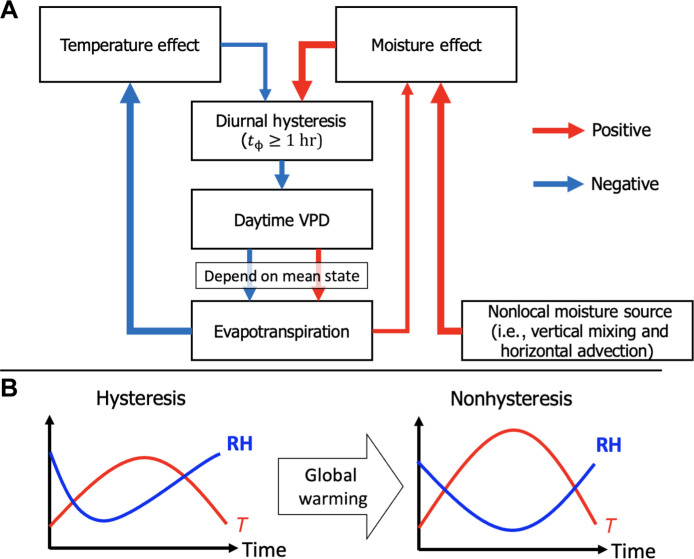
Schematic diagram of diurnal *T*/RH hysteresis at certain montane and coastal locations. (**A**) Mechanism of formatting diurnal *T*/RH hysteresis. Note that the red arrow is a positive effect, while the blue arrow indicates a negative effect. (**B**) Global warming impact on the diurnal *T*/RH hysteretic relationship. The red line represents the diurnal cycle of *T*, while the blue line depicts the diurnal cycle of RH.

Figure 7A summarizes the mechanisms driving the formation of diurnal *T*/RH hysteresis at certain montane and coastal regions. In the morning, rising *T* increases *Q*_s_, decreasing RH and inhibiting the development of diurnal *T*/RH hysteresis. Conversely, an increase in *Q* raises RH, promoting the formation of diurnal *T*/RH hysteresis. In the regions exhibiting diurnal *T*/RH hysteresis identified in Fig. 3, peak *T* occurs from 12:00 p.m. to 2:00 p.m., irrespective of the presence of diurnal *T*/RH hysteresis (Fig. 4, A to D, and fig. S2, A to D). However, areas exhibiting diurnal *T*/RH hysteresis show a larger *Q* range compared to adjacent areas. This substantial moisture effect can counteract the negative impact of *T* changes on RH in these regions (Fig. 5, E to H, and fig. S4, E to H), leading to an earlier occurrence of daily minimum RH. This interaction forms a distinct out-of-phase relationship between *T* and RH, which can affect local land-atmosphere interactions by altering VPD.

Diurnal *T*/RH hysteresis can mitigate VPD by both lowering overall VPD levels and altering the diurnal cycle (Fig. 4, M to P, and fig. S2, M to P). This reduction in daily VPD influences evapotranspiration patterns differently across various climates (see Supplementary Text). Furthermore, the alteration of the diurnal VPD cycle may influence the local carbon cycle. In regions exhibiting diurnal *T*/RH hysteresis, VPD reaches its daytime maximum before noon, leading to higher stomatal conductance at noon and thereby contributing to higher photosynthesis and carbon uptake (*3*, *4*, *7*, *34*). These findings highlight the potential implications of diurnal *T*/RH hysteresis on the terrestrial carbon cycle at certain montane and coastal regions.

The VPD mitigation effect induced by diurnal *T*/RH hysteresis can also influence the accuracy of daily VPD estimates, typically resulting in smaller underestimation errors compared to adjacent areas (Fig. 5, A to D). This underscores the importance of using subdaily data and challenges previous research that used daily or monthly datasets to study ecosystem responses to VPD, especially in regions with diurnal *T*/RH hysteresis (*34*–*38*). Many studies have relied on daytime mean *T* and RH to estimate daytime VPD. However, this approach may lead to underestimated VPD because of the nonlinear relationship between *T* and vapor pressure. While previous studies have developed various correction methods to estimate VPD (*9*, *35*), these methods typically assume an in-phase diurnal relationship between *T* and RH. However, in regions exhibiting diurnal *T*/RH hysteresis, such assumptions can lead to systematic overestimations of VPD, potentially exaggerating ecosystem sensitivity to atmospheric dryness. Moreover, these overestimations in regions with diurnal *T*/RH hysteresis may lead to an overestimation of potential evapotranspiration (*9*) and an underestimation of stomatal conductance (*12*, *39*), ultimately increasing uncertainty in evapotranspiration estimates in climate models. Therefore, revisiting global ecosystem responses to VPD using subdaily datasets is essential for accurately assessing VPD’s impact on land-atmosphere interactions.

Furthermore, although regions with diurnal *T*/RH hysteresis show a greater increase in morning *Q*, likely driven by local evapotranspiration and lateral water vapor transport, these areas usually exhibit lower morning evapotranspiration (Fig. 5, I to L, and fig. S4, I to L). This suggests that a nonlocal moisture source, such as vertical mixing and lateral water vapor transport, plays a role in increasing *Q* and contributing to the formation of diurnal *T*/RH hysteresis. Sources of this nonlocal moisture may include local mountain-valley winds and sea and lake breezes (*40*, *41*), which are common in areas where diurnal *T*/RH hysteresis is typically observed. While this study qualitatively underscores the pivotal role of lateral moisture transport in shaping diurnal *T*/RH hysteresis, quantifying the specific contributions of lateral moisture transport or local evapotranspiration to changes in *Q* remains a challenge. Further investigations, such as using model simulations and water isotope analysis (*42*, *43*), to unravel the complex interactions are essential.

Diurnal *T*/RH hysteresis, which mitigates VPD, is likely to diminish under global warming. Such changes in the diurnal *T*/RH hysteretic relationship could result in an increase in VPD. Previous studies have indicated that *Q*_s_ tends to rise more rapidly than *Q* under warming scenarios, contributing to an elevated VPD trend (*35*, *38*, *44*, *45*). Consequently, regions currently exhibiting diurnal *T*/RH hysteresis may face exacerbated VPD because of the impact of global warming on the diurnal *T*/RH hysteretic relationship (Fig. 7B). This increased VPD could suppress vegetation growth (*12*, *35*, *39*), increase tree mortality (*38*), and intensify heatwaves through land-atmosphere interactions (*46*). In addition, regions with diurnal *T*/RH hysteresis are pronounced in cloud forests and fog-dependent ecosystems. Changes in diurnal *T*/RH hysteretic relationship associated with global warming may influence local fog formation and land evaporative fluxes (*28*, *29*, *47*), thereby posing a threat to ecosystems adapted to the foggy surroundings (*31*). However, the mechanisms underlying the reduction in diurnal *T*/RH hysteresis under global warming and the contribution of increased VPD to extreme climate events or ecosystem impacts have not been fully explored in this study. Further analysis is necessary to investigate how global warming affects the diurnal cycle of *Q* and *Q*_s_ and its consequent impact on land-atmosphere interactions, particularly in regions currently exhibiting diurnal *T*/RH hysteresis.

In this study, we systematically investigate the global distribution of regions exhibiting diurnal *T*/RH hysteresis and assess its repercussion on local microclimates. Identifying these regions is crucial not only for guiding targeted regional studies on the effects of diurnal *T*/RH hysteresis on land-atmosphere interactions but also for understanding the moisture sources that sustain this hysteresis. Our findings also highlight the need to reevaluate previous studies that relied on daily or monthly datasets to estimate the impact of VPD on ecosystems and land-atmosphere interactions. While our analysis focuses on the influence of global warming on diurnal *T*/RH hysteresis, additional factors such as interannual variability and CO_2_ fertilization could also shape diurnal *T*/RH hysteresis and its relationship with VPD and evapotranspiration (*48*, *49*). Further research into these climatic and anthropogenic drivers across diverse regions will be essential for advancing our understanding of land-atmosphere interactions on a subdaily scale.

## MATERIALS AND METHODS

To explore the diurnal *T*/RH hysteresis around the globe, this study uses the diurnal cycle of meteorological variables from in situ observations, including HadISD (*21*–*23*) and flux tower datasets (*14*, *25*, *50*), and ERA5-Land reanalysis (*26*) with diurnal *T*/RH hysteretic metrics. In addition, PGW simulations with CLM5 (*27*) are used to examine the global warming impact on diurnal *T*/RH hysteresis.

### In situ data

The FLUXNET2015 dataset (*25*), collected from 206 in situ flux tower sites distributed around the globe, is used in this study. The meteorological variables are gap filled with the marginal distribution sampling method (*51*), which seeks similar meteorological conditions that are both physically and temporally close to the missing data points. The temporal resolution of the FLUXNET2015 data is either half-hourly or hourly. Details of the flux tower sites are provided in table S3. Moreover, TW-CLM (*14*, *52*) and TW-LHC (*50*) sites in Taiwan are also used in this study (fig. S1). TW-CLM is a cloud forest situated in the northeastern region of Taiwan, which is characterized by a mountain-valley circulation pattern, bringing warm and humid air to the forest during daytime. On the other hand, TW-LHC is a non–cloud-fog forest located in Central Taiwan. Half-hourly observations from TW-CLM (2008 to 2011) and TW-LHC (2009 to 2011) are used for the analysis.

Beside the flux tower dataset, HadISD version 3.3.1.202306p (last accessed 31 July 2023), which is retrieved from HadISD (*21*–*23*, *53*), is used in this study. The HadISD dataset is a global quality–controlled, subdaily, and weather station–based dataset, which contains a number of meteorological variables extended from 1931 to present. Because we focus on the overall diurnal pattern and mean diurnal *T*/RH hysteretic relationship, we do not apply homogeneity correction on each site and only use sites with hourly temporal resolution for analysis.

For the analysis of the diurnal hysteretic relationship between near-surface *T* and RH, we exclude sites lacking RH data or situated at latitudes above 60°. Sites with a temporal resolution exceeding 1 hour or with a mean daytime minimum *T* less than 0°C are also omitted. Ultimately, this study incorporates 182 flux tower sites and 4244 HadISD weather stations. We average the data in boreal summer (JJA) and boreal winter (DJF) by half-hourly or hourly intervals, depending on the temporal resolution of each site, for further diurnal hysteretic analysis.

### Reanalysis data

This study leverages the ERA5-Land dataset, a global land reanalysis product generated from the European Centre for Medium-Range Weather Forecasts (ECMWF). ERA5-Land is driven by downscaled meteorological forcing from ERA5 with the ECMWF land surface model, offering a finer spatial resolution of 0.1° by 0.1° and more accurate land variables (*26*). Specifically, we use the ERA5-Land monthly averaged reanalysis by hour of day, which is accessible through the Copernicus Climate Change Service (C3S) Climate Data Store (2022). This resource provides comprehensive and consistent diurnal patterns over several decades on a global scale.

For our analysis, we extract meteorological variables such as *T*, dew point temperature (*T*_d_), and surface pressure (*P*) for the period from 1951 to 2020 within the latitude range of 60°S to 60°N. These variables are then used to calculate RH, specific humidity (*Q*), saturated specific humidity (*Q*_s_), and VPD. With its hourly temporal resolution, ERA5-Land data are averaged by hour of day to delineate the diurnal cycle during JJA and DJF across each grid point.

### Model simulations

We use CLM5 to investigate the impact of global warming on diurnal *T*/RH hysteresis in the Andes with a resolution of 0.1° by 0.1°. The control run (CTR) is driven by ERA5 meteorological forcings (*54*) from 2000 to 2010, comparable to those of ERA5-Land.

To assess the effects of global warming on diurnal *T*/RH hysteresis, we conduct PGW simulations by adding future changes in the diurnal cycles of *Q* and *Q*_s_ to the CTR forcing. These future changes are derived from the anomalies from the historical period from 1986 to 2005 and projected future periods under global mean surface *T* increases of 1.5 and 3.0 K under the Representative Concentration Pathway 8.5 scenario. These projections are based on RCM simulations within the CORDEX-CORE framework (*55*) for South American regions, driven by various Coupled Model Intercomparison Project Phase 5 (CMIP5) global climate models. Two global warming scenarios are applied to PGW with a total of six RCM simulations (table S2). The output of PGW simulations is then averaged in an hourly interval in JJA and DJF for further diurnal hysteretic analysis.

### Diurnal hysteretic metrics

The method used for quantifying the diurnal hysteretic pattern has been extensively described in the previous literature (*6*, *56*), so here, we provide a brief overview for efficacy. Assuming that the diurnal cycle of RH and *T* can be approximated by harmonic functions, the diurnal cycle of RH can be decomposed into a response of *T* and its time derivative, shown in Eq. 1. This form is similar to the Bernardi-Camuffo formula (*57*), which was originally used to describe phase shifts between solar radiation and heat fluxes. By applying multilinear regression, we estimate the coefficients $\beta_{1}$ and $\beta_{2}$ in Eq. 1. If RH is linear to *T*, meaning no diurnal hysteresis between *T* and RH, $\beta_{2}$ should be zero. Conversely, a nonzero $\beta_{2}$ indicates the presence of diurnal hysteresis between *T* and RH. Assuming that the diurnal cycle of *T* follows a harmonic function with angular frequency ω , the phase lag between *T* and RH can be estimated using Eq. 2, expressed in hourly units. Positive (negative) values of $t_{\phi }$ denote that RH precedes (lag behind) changes in *T*.

$$\text{RH}\left(t\right)=\beta_{1}T\left(t\right)+\beta_{2}\frac{\partial }{\partial t}T\left(t\right)+\beta_{0}+\epsilon \left(t\right)$$ (1)

where RH(t) is the diurnal cycle of RH, T(t) is the diurnal cycle of *T*, and $\frac{\partial }{\partial t}T\left(t\right)$ is the diurnal cycle of time deviation of *T*. $\beta_{1},\beta_{2},$ and $\beta_{0}$ are the coefficients in multilinear regression, and ϵ(t) is the residual term.

$$t_{\phi }={\text{tan}}^{-1}\left(\frac{\beta_{2}\omega }{\beta_{1}}\right)\frac{24}{2\pi }={\text{tan}}^{-1}\left(\frac{\beta_{2}}{\beta_{1}}\frac{2\pi }{n_{d}}\right)\frac{24}{2\pi }\;$$ (2)

where $t_{\phi }$ is the phase lag time between *T* and RH; $\beta_{1}$ , $\beta_{2}$ , and $\beta_{0}$ are the coefficients in multilinear regression accessed in Eq. 1, and $n_{d}$ is the number of time steps per day.

We also compared this method with a simpler approach that estimates the phase lag on the basis of the local solar times of *T* maximum and RH minimum. Our analysis shows that the two approaches generally produce consistent results; however, notable differences arise in regions exhibiting strong hysteresis signals. These discrepancies primarily result from slight differences in the angular frequencies of *T* and RH, as well as the presence of sites without distinct diurnal cycles, which can lead to the difference in the estimated phase lag when using the peak-time approach compared to the regression-based estimate (figs. S5 and S6).

We use the diurnal cycle from 6:00 a.m. to 6:00 p.m. with 3-hourly running mean of *T* and RH to access $t_{\phi }$ . The diurnal cycle in which the daytime minimum *T* is lower than 0°C is removed. The diurnal cycles in which the *P* value of $\beta_{0}$ , $\beta_{1}$ , and $\beta_{2}$ in the multilinear regression is larger than 0.05 are also removed. As most of the time resolution of the dataset, which we use in this study, is hourly, we define regions exhibiting diurnal *T*/RH hysteresis as $t_{\phi }$ larger than 1 hour.

### Bias in mean daytime VPD estimation

VPD is defined as the difference between saturated vapor pressure and actual vapor pressure. Estimations of VPD often underestimate its true value when derived from daytime mean *T* and RH, primarily due to the lack of subdaily meteorological data. In this study, we use Eq. 3 to quantify the bias in mean daytime VPD estimations from 6:00 a.m. to 6:00 p.m., where a negative value indicates an underestimation. This underestimation arises from the nonlinear relationship between *T* and saturated vapor pressure based on the Clausius-Clapeyron relationship.

$$\epsilon =\frac{\text{VP}D_{\bar{T},\bar{\text{RH}}}-\bar{\text{VPD}}}{\bar{\text{VPD}}}$$ (3)

where ϵ is the bias in mean daytime VPD estimation, $\text{VP}D_{\bar{T},\bar{\text{RH}}}$ is daytime VPD estimated by daytime mean *T* and RH, and $\bar{\text{VPD}}$ is daytime VPD estimated by each time step of VPD.

### Proofreading with the assistance of ChatGPT

Portions of the English writing were proofread using OpenAI’s ChatGPT (GPT-4o) to improve clarity and readability while maintaining scientific accuracy. An example prompt used during proofreading was the following

“Please proofread below content: Although this phenomenon is found in less than 5% of all observation sites (combining HadISD and flux tower data), with only 1.46% of stations in the HadISD dataset exhibiting it, this may be largely due to the fact that most weather stations are not located in the montane and coastal regions, where ERA5-Land data indicate its presence.”

ChatGPT provided the following proofread version

“Your statement is already strong, but here’s a refined version with improved readability and flow while maintaining emphasis on the rarity of the phenomenon: ‘This phenomenon is observed in less than 5% of all observation sites, combining HadISD and flux tower data, with only 1.46% of stations in the HadISD dataset exhibiting it. This limited occurrence may be largely due to the fact that most weather stations are not located in the montane and coastal regions, where ERA5-Land data indicate its presence.’”

The artificial intelligence–assisted proofreading was limited to linguistic refinements and did not alter the scientific content or conclusions.
