# Supplementary material for: Impact of diurnal temperature and relative humidity hysteresis on atmospheric dryness in changing climates
Source: Sci Adv. 2025 Jun 27;11(26):eadu5713. doi: 10.1126/sciadv.adu5713 (PMC12204172; doi:10.1126/sciadv.adu5713)
Supplement: Supplementary file 1 — Supplementary Text Figs. S1 to S6 Tables S1 to S3 [file sciadv.adu5713_sm.pdf]

Science Advances
AAAS

Supplementary Materials for

Impact of diurnal temperature and relative humidity hysteresis on atmospheric dryness in changing climates

Ching-Hung Shih *et al.*

Corresponding author: Min-Hui Lo, minhuilo@ntu.edu.tw

Sci. Adv. **11**, eadu5713 (2025)
DOI: 10.1126/sciadv.adu5713

This PDF file includes:

Supplementary Text
Figs. S1 to S6
Tables S1 to S3

Supplementary Text

Supplementary text 1 - Impact of Diurnal T/RH Hysteresis on VPD and Evapotranspiration

Despite diurnal T/RH hysteresis consistently mitigating VPD across these regions, the response of the evaporative fraction (EF), defined as the ratio of evapotranspiration to net radiation, varies (Fig. S3). In humid areas like New Guinea and the Himalayas, where mean VPD ranges from 0 to 1 kPa, EF and VPD show a positive correlation. Conversely, in drier regions such as the Andes and southwestern Arabia, where mean VPD ranges from 0 to 6 kPa, the correlation between EF and VPD is negative. This diversity in evapotranspiration responses suggests that while diurnal T/RH hysteresis uniformly mitigates VPD, its impact on evapotranspiration differs across climatic regions: humid regions exhibit higher evapotranspiration, whereas drier regions experience lower evapotranspiration compared to adjacent areas (Fig. 5I-L; Fig. S4I-L).

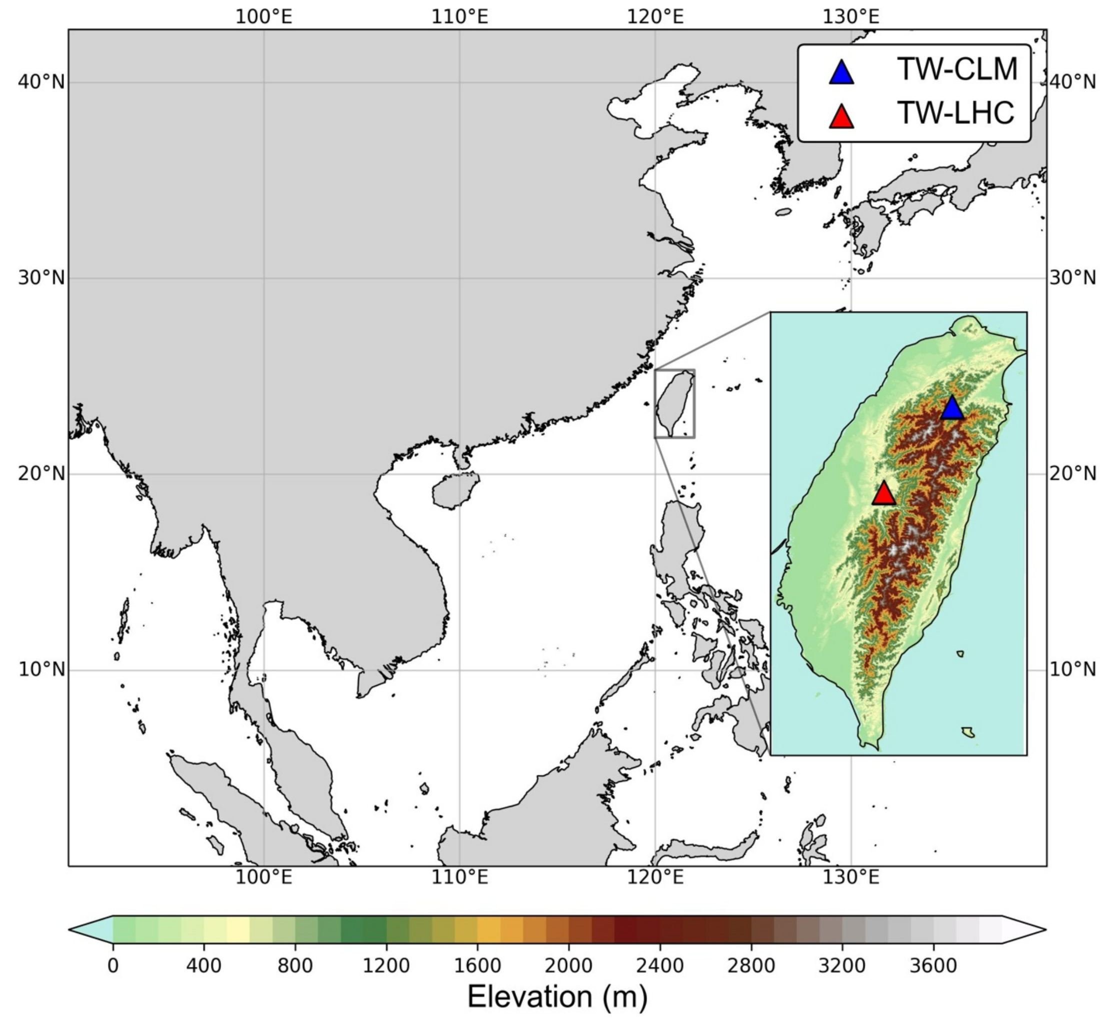

Fig. S1. The locations of Chi-Lan (TW-CLM) and Lien-Hua-Chih (TW-LHC) sites in Taiwan.

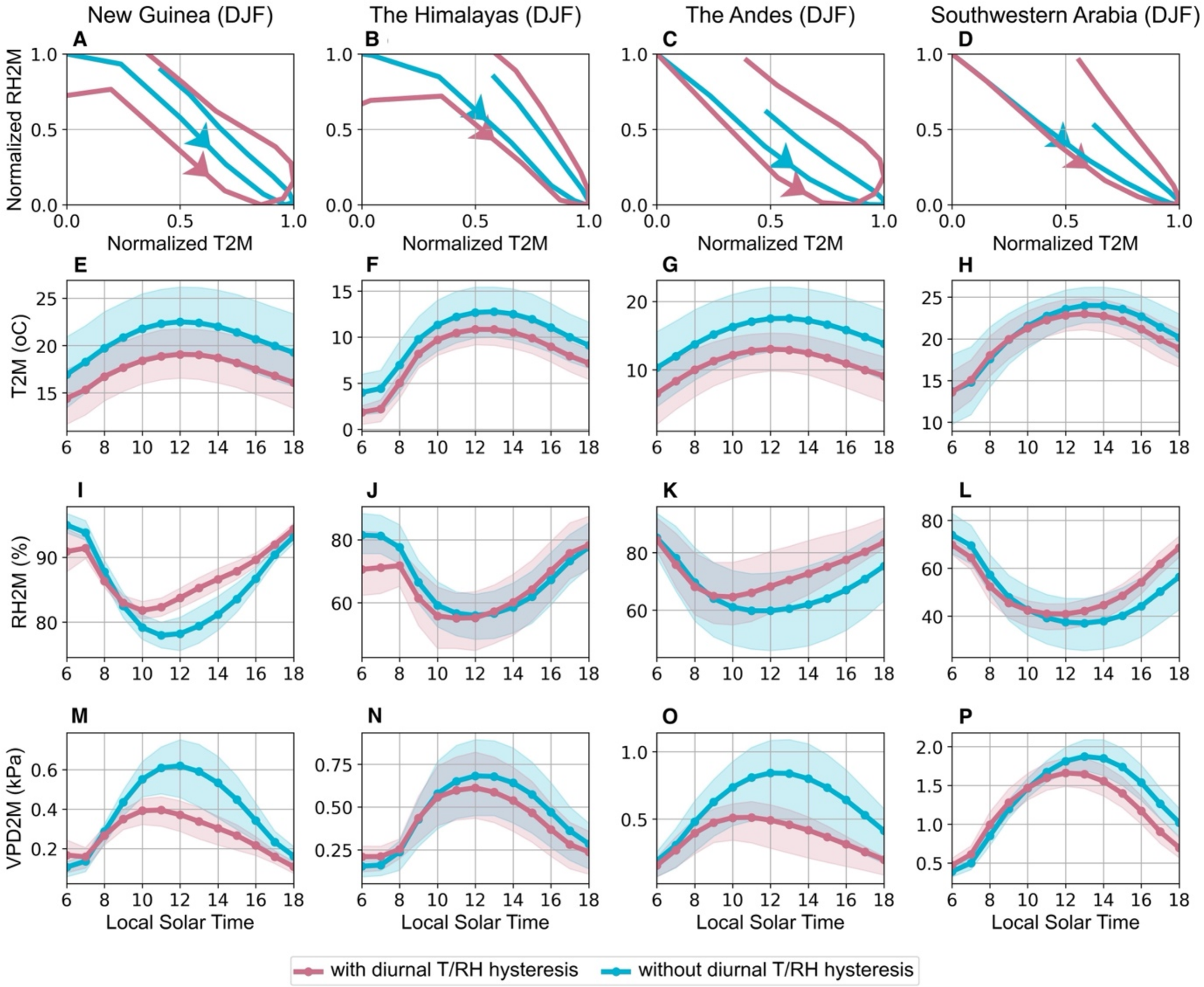

Fig. S2. The diurnal cycles in DJF from 1951 to 2020 in areas exhibiting diurnal T/RH hysteresis (red lines) and adjacent areas without diurnal T/RH hysteresis (blue lines), across the four diurnal T/RH hysteresis regions identified in Fig. 3. (**A-D**) depict the diurnal phase diagram between normalized T and normalized RH. All hysteretic cycles are counter-clockwise. (**E-H**), (**I-L**), and (**M-P**) illustrate the diurnal cycle of 2-meter T (T2M; °C), 2-meter RH (RH2M; %), and 2-meter VPD (VPD2M; kPa), respectively. The shadings represent the range of variation of each meteorological variable between the first and the third quartiles of data. It is noteworthy that the surrounding areas without diurnal T/RH hysteresis (blue lines) were selected through buffer analysis. This analysis ensured that the number of grid cells without diurnal T/RH hysteresis approximates the number of those with diurnal T/RH hysteresis, facilitating a balanced comparison between regions with and without this phenomenon. Note that the mean diurnal curves are derived by spline fitting to the hourly data.

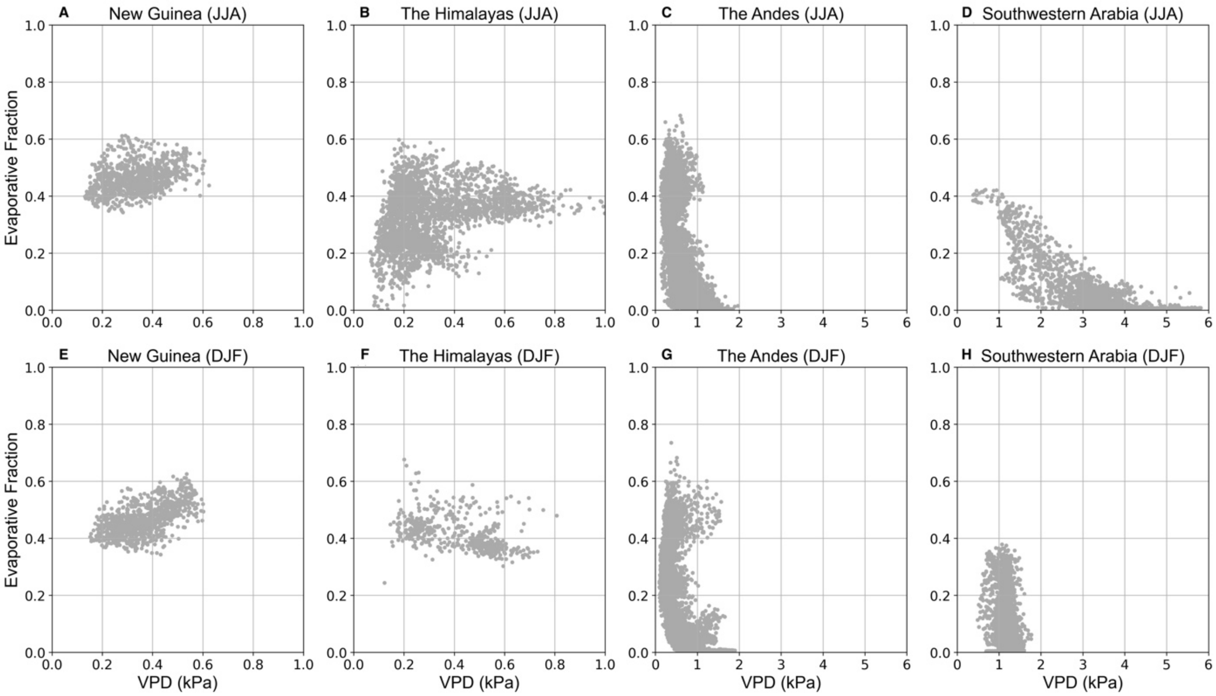

Fig. S3. The evaporative fraction-VPD relationship from 1951 to 2020. (A-D) and (E-H) depict the presence in JJA and DJF, respectively, in areas exhibiting diurnal T/RH hysteresis and adjacent areas without diurnal T/RH hysteresis across the four primary hysteresis regions identified in Figure 2.

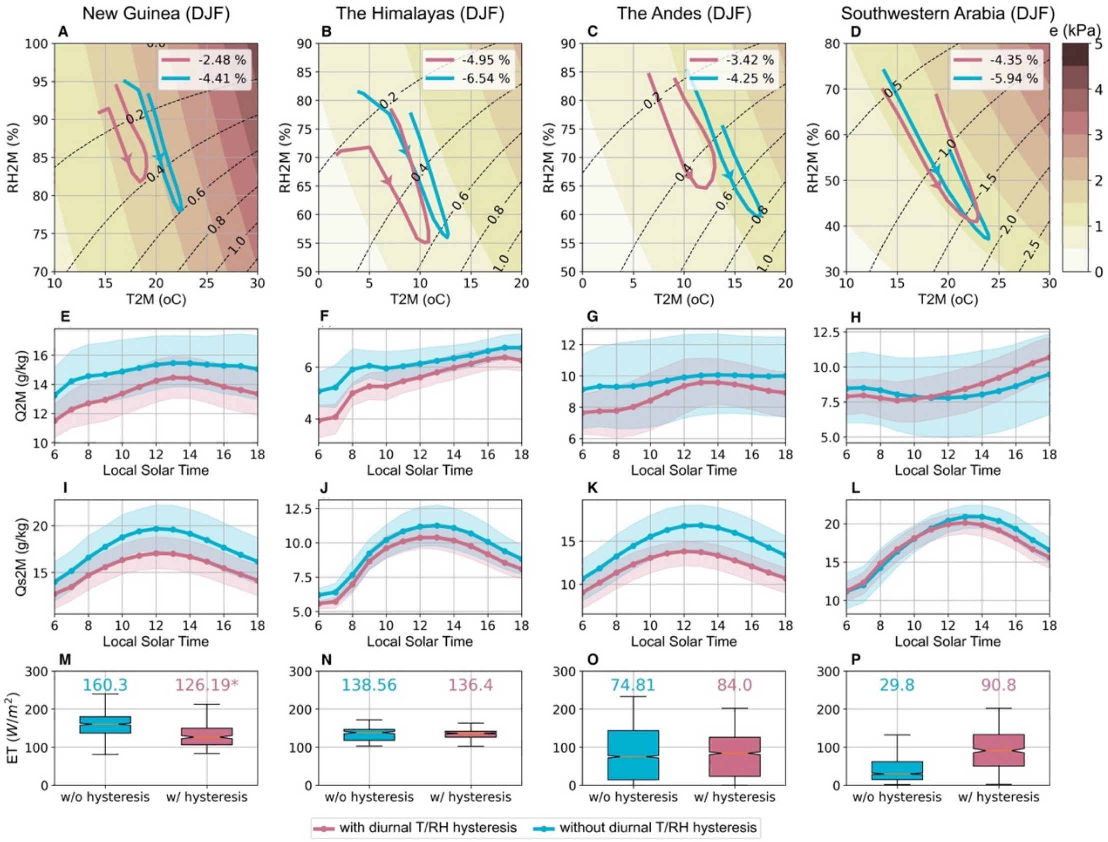

Fig. S4. The comparison between areas exhibiting diurnal T/RH hysteresis (red) and adjacent areas without diurnal T/RH hysteresis (blue) in DJF from 1951 to 2020, across the four diurnal T/RH hysteresis regions identified in Fig. 3. (**A-D**) depict the regional mean of diurnal phase diagram between 2-meter T (T2M) and 2-meter RH (RH2M). The shading within each panel indicates vapor pressure (kPa), while black dashed lines denote VPD (kPa). The legends quantify the bias in mean daytime (from 6 a.m. to 6 p.m.) VPD estimation (%). All hysteretic cycles are counter-clockwise. (**E-H**) and (**I-L**) illustrate the diurnal cycle of 2-meter specific humidity (Q2M; g/kg) and 2-meter saturated specific humidity (Qs2M; g/kg), respectively. The shadings represent the range of variation of each meteorological variable between the first and the third quartiles of data. (**M-P**) represent the comparison of evapotranspiration (W/m^2) from 6 am to noon. The numbers above the boxes denote the median values. The presence of asterisks evapotranspiration is significantly lower in these hysteresis regions. These findings are supported by Student's t-tests, with asterisks denoting p-values smaller than 0.05 (*). (see Materials and Methods for the definition of bias in mean daytime VPD estimation). Note that the mean diurnal curves in Fig. 5 **E-L** are derived by spline fitting to the hourly data.

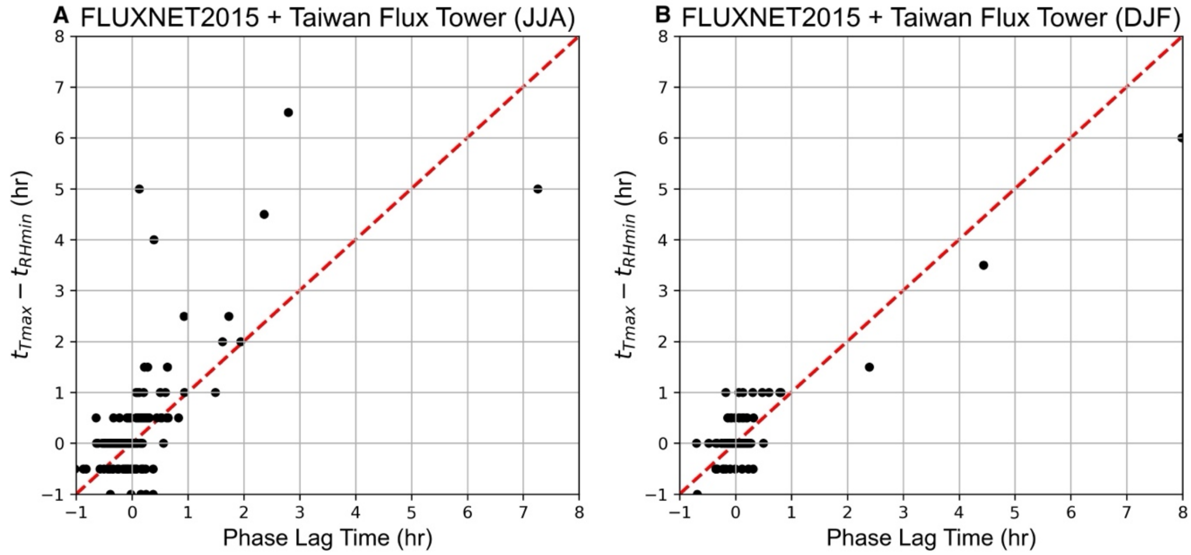

Fig. S5. Comparison of two different methods quantifying diurnal T/RH hysteresis with FLUXNET2015 and Taiwan flux tower dataset. (A) and (B) represent scatter plots showing the comparison between phase lag time (hr), which is calculated by Eqs. 1–2, and the difference between maximum T and minimum RH (hr).

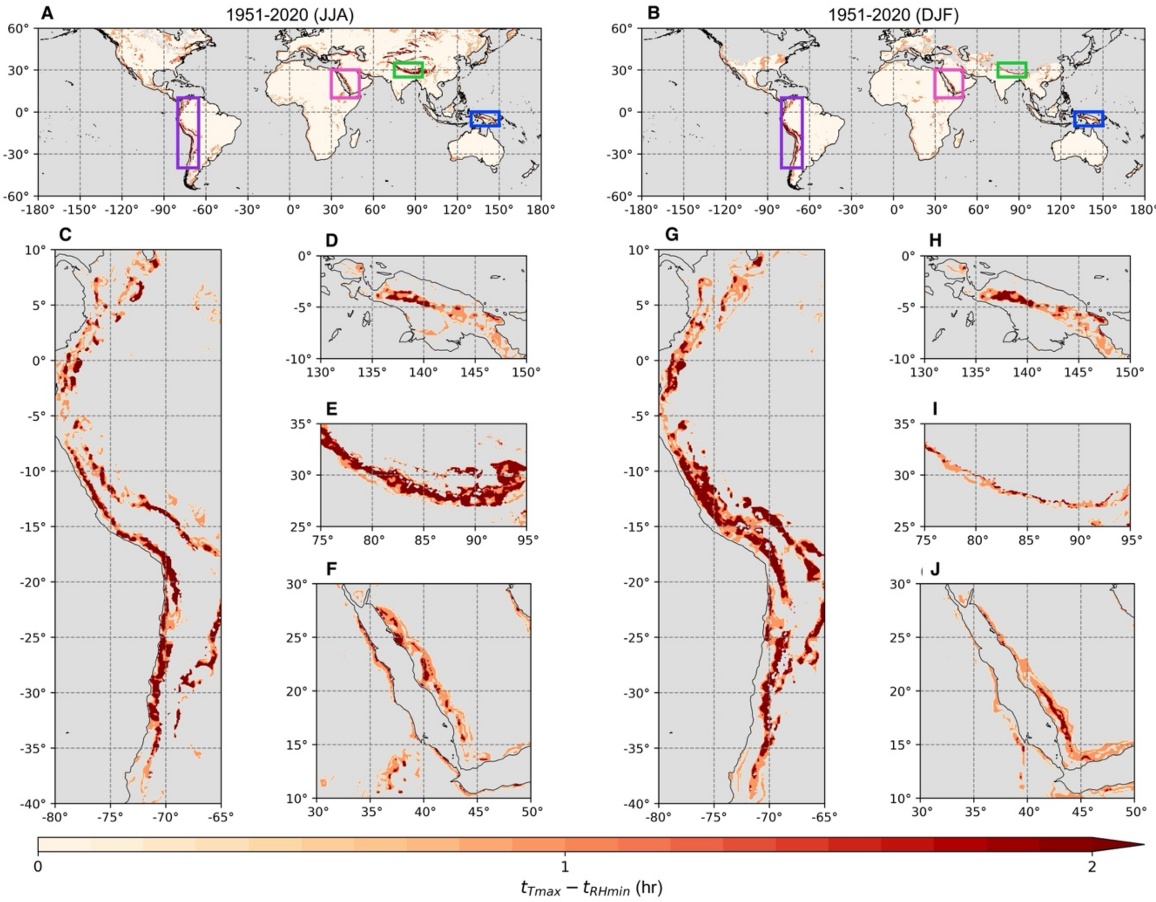

Fig. S6 The presence of diurnal T/RH hysteresis in ERA5-Land from 1951 to 2020. (**A**, **C**, **D**, **E** and **F**) and (**B**, **G**, **H**, **I** and **J**) depict the presence in JJA and DJF, respectively. The boxes illustrate the main four regions exhibiting diurnal T/RH hysteresis, including the Andes (purple boxes; **C** and **G**), New Guinea (blue boxes; **D** and **H**), the Himalayas (green boxes; **E** and **I**), and Southwestern Arabia (pink boxes; **F** and **J**). The red monochromatic colors in all panels represent the time difference between maximum T and minimum RH (hr). The grey shadings represent the grids that were masked due to significant test and temperature lower than 0 °C. The green-brown-white color gradient in (**C-J**) represents the elevation. Note that only grids exhibiting diurnal T/RH hysteresis, defined as those where time difference exceeds one hour, are shown in (**C**)-(**J**).

Table S1. The trend of phase lag time (hr/70yr) from 1951 to 2020 in the four regions with phase lag time larger than one hour in the 1950s, which is shown in Fig. 3AB. Statistical significance (Student's *t*-test) is indicated by the presence of asterisks, with stars indicating *p*-values smaller than 0.001 (***), 0.01 (**), or 0.05 (*).

	New Guinea		The Andes		The Himalayas		Southwestern Arabia	
	JJA	DJF	JJA	DJF	JJA	DJF	JJA	DJF
Phase lag time	-0.01	-0.01	-0.18***	-0.07***	-0.05	0.09*	-0.14***	0.05

Table S2. Description of regional climate models used for pseudo global warming (PGW) simulations.

RCM	Driving GCM
REMO2015 (GERICS)	HadGEM2-ES (MOHC)
	NorESM1-M (NCC)
	MPI-ESM-LR (MPI)
RegCM4-7 (ICTP)	HadGEM2-ES (MOHC)
	NorESM1-M (NCC)
	MPI-ESM-MR (MPI)

Table S3. Information on the 208 flux tower sites from the FLUXNET2015 datasets. Site identifier (ID), study periods, latitude (°), longitude (°) and elevation (m) are listed.

Site ID	year_start	year_end	Longitude	Latitude	Elevation
AR-SLu	2009	2011	-66.4598	-33.4648	713
AR-Vir	2009	2012	-56.1886	-28.2395	9.45
AT-Neu	2002	2012	11.3175	47.11667	970
AU-ASM	2010	2014	133.249	-22.283	61.7
AU-Ade	2007	2009	131.1178	-13.0769	7.51
AU-Cpr	2010	2014	140.5891	-34.0021	4.81
AU-Cum	2012	2014	150.72362	-33.61518	8.51
AU-DaP	2007	2013	131.3181	-14.0633	6.51
AU-DaS	2008	2014	131.3881	-14.1593	7.62
AU-Dry	2008	2014	132.3706	-15.2588	17.75
AU-Emr	2011	2013	148.4746	-23.8587	16.4
AU-Fog	2006	2008	131.3072	-12.5452	0.37
AU-GWW	2013	2014	120.6541	-30.1913	46.1
AU-Gin	2011	2014	115.7138	-31.3764	4.89
AU-How	2001	2014	131.1523	-12.4943	2.37
AU-Lox	2008	2009	140.6551	-34.4704	4.72
AU-RDF	2011	2013	132.4776	-14.5636	18.57
AU-Rig	2011	2014	145.5759	-36.6499	13.65
AU-Rob	2014	2014	145.6301	-17.1175	71.71
AU-Stp	2008	2014	133.3502	-17.1507	22.61
AU-TTE	2012	2014	133.64	-22.287	57.09
AU-Tum	2001	2014	148.1517	-35.6566	1200
AU-Wac	2005	2008	145.1878	-37.4259	51.22
AU-Whr	2011	2014	145.0294	-36.6732	16.48
AU-Wom	2010	2014	144.0944	-37.4222	705
AU-Ync	2012	2014	146.2907	-34.9893	12.7
BE-Bra	1996	2014	4.51984	51.30761	16
BE-Lon	2004	2014	4.74623	50.55162	167

BE-Vie	1996	2014	5.99812	50.30493	493
BR-Sa1	2002	2011	-54.95889	-2.85667	88
BR-Sa3	2000	2004	-54.97144	-3.01803	100
CA-Gro	2003	2014	-82.1556	48.2167	340
CA-Man	1994	2008	-98.48081	55.87962	259
CA-NS1	2001	2005	-98.48389	55.87917	260
CA-NS2	2001	2005	-98.52472	55.90583	260
CA-NS3	2001	2005	-98.38222	55.91167	260
CA-NS4	2002	2005	-98.380645	55.91437	260
CA-NS5	2001	2005	-98.485	55.86306	260
CA-NS6	2001	2005	-98.96444	55.91667	244
CA-NS7	2002	2005	-99.94833	56.63583	297
CA-Oas	1996	2010	-106.19779	53.62889	530
CA-Obs	1997	2010	-105.11779	53.98717	628.94
CA-Qfo	2003	2010	-74.34206	49.6925	383
CA-SF1	2003	2006	-105.81757	54.48503	536
CA-SF2	2001	2005	-105.8775	54.25392	520
CA-SF3	2001	2006	-106.00526	54.09156	540
CA-TP1	2002	2014	-80.559519	42.6609361	265
CA-TP2	2002	2007	-80.458775	42.7744194	212
CA-TP3	2002	2014	-80.348314	42.7068111	184
CA-TP4	2002	2014	-80.357376	42.710161	184
CA-TPD	2012	2014	-80.557731	42.635328	260
CG-Tch	2006	2009	11.65642	-4.28917	82
CH-Cha	2005	2014	8.41044	47.21022	393
CH-Dav	1997	2014	9.85591	46.81533	1639
CH-Fru	2005	2014	8.53778	47.11583	982
CH-Lae	2004	2014	8.36439	47.47833	689
CH-Oe1	2002	2008	7.73194	47.28583	450
CH-Oe2	2004	2014	7.73375	47.28642	452
CN-Cha	2003	2005	128.0958	42.4025	81.54

CN-Cng	2007	2010	123.5092	44.5934	14.74
CN-Dan	2004	2005	91.0664	30.4978	482
CN-Din	2003	2005	112.5361	23.1733	23.7
CN-Du2	2006	2008	116.2836	42.0467	146.6
CN-Du3	2009	2010	116.2809	42.0551	141.22
CN-Ha2	2003	2005	101.3269	37.6086	322.5
CN-HaM	2002	2004	101.18	37.37	378.5
CN-Qia	2003	2005	115.0581	26.7414	13.6
CN-Sw2	2010	2012	111.8971	41.7902	149
CZ-BK1	2004	2014	18.53688	49.50208	875
CZ-BK2	2004	2012	18.54285	49.49443	855
CZ-wet	2006	2014	14.77035	49.02465	426
DE-Akm	2009	2014	13.68342	53.86617	-1
DE-Geb	2001	2014	10.91463	51.09973	161.5
DE-Gri	2004	2014	13.51253	50.94947	385
DE-Hai	2000	2012	10.45217	51.07921	430
DE-Kli	2004	2014	13.52238	50.89306	478
DE-Lkb	2009	2013	13.30467	49.09962	1308
DE-Lnf	2002	2012	10.3678	51.32822	451
DE-Obe	2008	2014	13.72129	50.78666	734
DE-RuR	2011	2014	6.30413	50.62191	514.7
DE-RuS	2011	2014	6.44717	50.86591	102.8
DE-Seh	2007	2010	6.44965	50.87062	103
DE-SfN	2012	2014	11.3275	47.80639	590
DE-Spw	2010	2014	14.03369	51.89225	61
DE-Tha	1996	2014	13.56515	50.96256	385
DE-Zrk	2013	2014	12.88901	53.87594	0
DK-Eng	2005	2008	12.19175	55.69053	10
DK-Fou	2005	2005	9.58722	56.4842	51
DK-Sor	1996	2014	11.64464	55.48587	40
ES-Amo	2007	2012	-2.25232	36.83361	58

ES-LJu	2004	2013	-2.75212	36.92659	1600
ES-LgS	2007	2009	-2.96583	37.09794	2267
ES-Ln2	2009	2009	-3.47582	36.9695	2249
FI-Hyy	1996	2014	24.29477	61.84741	181
FI-Jok	2000	2003	23.51345	60.8986	109
FI-Let	2009	2012	23.95952	60.64183	111
FI-Lom	2007	2009	24.20918	67.99724	274
FI-Sod	2001	2014	26.63859	67.36239	180
FR-Fon	2005	2014	2.7801	48.47636	103
FR-Gri	2004	2014	1.95191	48.84422	125
FR-LBr	1996	2008	-0.7693	44.71711	61
FR-Pue	2000	2014	3.5957	43.7413	270
GF-Guy	2004	2014	-52.92486	5.27877	48
GH-Ank	2011	2014	-2.69421	5.26854	124
GL-NuF	2008	2014	-51.38611	64.13083	50
GL-ZaF	2008	2011	-20.55452	74.48143	38
GL-ZaH	2000	2014	-20.5503	74.47328	38
IT-BCi	2004	2014	14.95744	40.52375	20
IT-CA1	2011	2014	12.02656	42.38041	200
IT-CA2	2011	2014	12.02604	42.37722	200
IT-CA3	2011	2014	12.0222	42.38	197
IT-Col	1996	2014	13.58814	41.84936	1560
IT-Cp2	2012	2014	12.35729	41.70427	19
IT-Cpz	1997	2009	12.37611	41.70525	68
IT-Isp	2013	2014	8.63358	45.81264	210
IT-La2	2000	2002	11.2853	45.9542	1350
IT-Lav	2003	2014	11.28132	45.9562	1353
IT-MBo	2003	2013	11.04583	46.01468	1550
IT-Noe	2004	2014	8.15169	40.60618	25
IT-PT1	2002	2004	9.06104	45.20087	60
IT-Ren	1998	2013	11.43369	46.58686	1730

IT-Ro1	2000	2008	11.93001	42.40812	235
IT-Ro2	2002	2012	11.92093	42.39026	160
IT-SR2	2013	2014	10.29091	43.73202	4
IT-SRo	1999	2012	10.28444	43.72786	6
IT-Tor	2008	2014	7.57806	45.84444	2160
JP-MBF	2003	2005	142.3186	44.3869	585
JP-SMF	2002	2006	137.0788	35.2617	205
MY-PSO	2003	2009	102.3062	2.973	100
NL-Hor	2004	2011	5.0713	52.24035	2.2
NL-Loo	1996	2014	5.74356	52.16658	25
PA-SPn	2007	2009	-79.6346	9.31814	78
PA-SPs	2007	2009	-79.63143	9.31378	68
RU-Che	2002	2005	161.34143	68.61304	6
RU-Cok	2003	2014	147.49428	70.82914	48
RU-Fyo	1998	2014	32.92208	56.46153	265
RU-Ha1	2002	2004	90.00215	54.72517	446
SD-Dem	2005	2009	30.4783	13.2829	500
SJ-Adv	2011	2014	15.923	78.186	17
SJ-Blv	2008	2009	11.83109	78.92163	25
SN-Dhr	2010	2013	-15.43222	15.40278	40
US-AR1	2009	2012	-99.42	36.4267	611
US-AR2	2009	2012	-99.5975	36.6358	646
US-ARM	2003	2012	-97.4888	36.6058	314
US-ARb	2005	2006	-98.0402	35.5497	424
US-ARc	2005	2006	-98.04	35.54649	424
US-Atq	2003	2008	-157.4089	70.4696	15
US-Blo	1997	2007	-120.6328	38.8953	1315
US-CRT	2011	2013	-83.347086	41.628495	180
US-Cop	2001	2007	-109.39	38.09	1520
US-GBT	1999	2006	-106.2397	41.36579	3191
US-GLE	2004	2014	-106.2399	41.36653	3197

US-Goo	2002	2006	-89.8735	34.2547	87
US-Ha1	1991	2012	-72.1715	42.5378	340
US-IB2	2004	2011	-88.24103	41.84062	226.5
US-Ivo	2004	2007	-155.7503	68.4865	568
US-KS1	2002	2002	-80.6709	28.4583	1
US-KS2	2003	2006	-80.6715	28.6086	3
US-LWW	1997	1998	-97.9789	34.9604	365
US-Lin	2009	2010	-119.8423	36.3566	131
US-Los	2000	2014	-89.9792	46.0827	480
US-MMS	1999	2014	-86.4131	39.3232	275
US-Me1	2004	2005	-121.5	44.5794	896
US-Me2	2002	2014	-121.5574	44.4523	1253
US-Me3	2004	2009	-121.6078	44.3154	1005
US-Me4	1996	2000	-121.6224	44.4992	922
US-Me5	2000	2002	-121.56676	44.43719	1188
US-Me6	2010	2014	-121.6078	44.3232842	998
US-Myb	2010	2014	-121.76506	38.049782	-4
US-NR1	1998	2014	-105.5464	40.0329	3050
US-Ne1	2001	2013	-96.47664	41.16506	361
US-Ne2	2001	2013	-96.4701	41.16487	362
US-Ne3	2001	2013	-96.43965	41.17967	363
US-ORv	2011	2011	-83.0183	40.0201	221
US-Oho	2004	2013	-83.8438	41.5545	230
US-PFa	1995	2014	-90.2723	45.9459	470
US-Prr	2010	2014	-147.48756	65.12367	210
US-SRC	2008	2014	-110.8395	31.9083	950
US-SRG	2008	2014	-110.82768	31.789379	1291
US-SRM	2004	2014	-110.8661	31.8214	1120
US-Sta	2005	2009	-106.8024	41.3966	2069
US-Syv	2001	2014	-89.3477	46.242	540
US-Ton	2001	2014	-120.96598	38.4316	177

US-Tw1	2012	2014	-121.6469	38.1074	-5
US-Tw2	2012	2013	-121.6433	38.1047	-5
US-Tw3	2013	2014	-121.6467	38.1159	-4
US-Tw4	2013	2014	-121.6414	38.10298	-5
US-Twt	2009	2014	-121.6531	38.1087204	-7
US-UMB	2000	2014	-84.7138	45.5598	234
US-UMd	2007	2014	-84.6975	45.5625	239
US-Var	2000	2014	-120.9507	38.4133	129
US-WCr	1999	2014	-90.0799	45.8059	520
US-WPT	2011	2013	-82.996157	41.464639	175
US-Whs	2007	2014	-110.0522	31.7438	1370
US-Wi0	2002	2002	-91.081444	46.618778	349
US-Wi1	2003	2003	-91.232944	46.730472	352
US-Wi2	2003	2003	-91.152833	46.686889	395
US-Wi3	2002	2004	-91.098667	46.634722	411
US-Wi4	2002	2005	-91.16625	46.739333	352
US-Wi5	2004	2004	-91.085806	46.653083	353
US-Wi6	2002	2003	-91.298222	46.624889	371
US-Wi7	2005	2005	-91.069278	46.649111	335
US-Wi8	2002	2002	-91.252417	46.722333	348
US-Wi9	2004	2005	-91.081444	46.618778	350
US-Wkg	2004	2014	-109.9419	31.7365	1531
ZM-Mon	2000	2009	23.2525	-15.4391	1053
